# Another tool against cytomegalovirus after allogeneic hematopoietic cell transplantation

**DOI:** 10.1172/JCI170282

**Published:** 2023-05-15

**Authors:** George L. Chen, Elizabeth J. Shpall

**Affiliations:** Stem Cell Transplantation and Cellular Therapy, The University of Texas MD Anderson Cancer Center, Houston, Texas, USA.

## Abstract

Cytomegalovirus (CMV) viremia from reactivation of latent infection is a common complication after allogeneic hematopoietic cell transplantation (HCT). Untreated, CMV viremia can progress to affect other organs, resulting in organ dysfunction with high morbidity and mortality. In this issue of the *JCI*, Prockop and authors demonstrate that third-party donor T cells sensitized ex vivo to CMV pp65-derived overlapping pentadecapeptides are safe and effective for the treatment of CMV reactivation or CMV disease refractory to first-line pharmacotherapies occurring after HCT. They also provide insight into the biological differences between responders and nonresponders. This work confirms the utility of third-party CMV pp65 VSTs and suggests strategies for further improving the efficacy of this cell-therapy approach.

## CMV is a persistent problem after HCT

Cytomegalovirus (CMV), a member of the herpesvirus family, is an enveloped, icosahedral capsid, double-stranded deoxyribonucleic acid virus. Based upon seroprevalence data, approximately 83% of the world’s population has been infected (1).

The human immune system devotes a large proportion of its resources to preventing and controlling CMV reactivation. In normal humans, up to 10% or more of the CD4^+^ and CD8^+^ memory T cell compartments are specific to CMV (2). In patients with chronic lymphocytic leukemia, as many as 37.7% of CD4^+^ T cells and 14% of CD8^+^ T cells are specific to CMV (3, 4).

**Figure 1:**
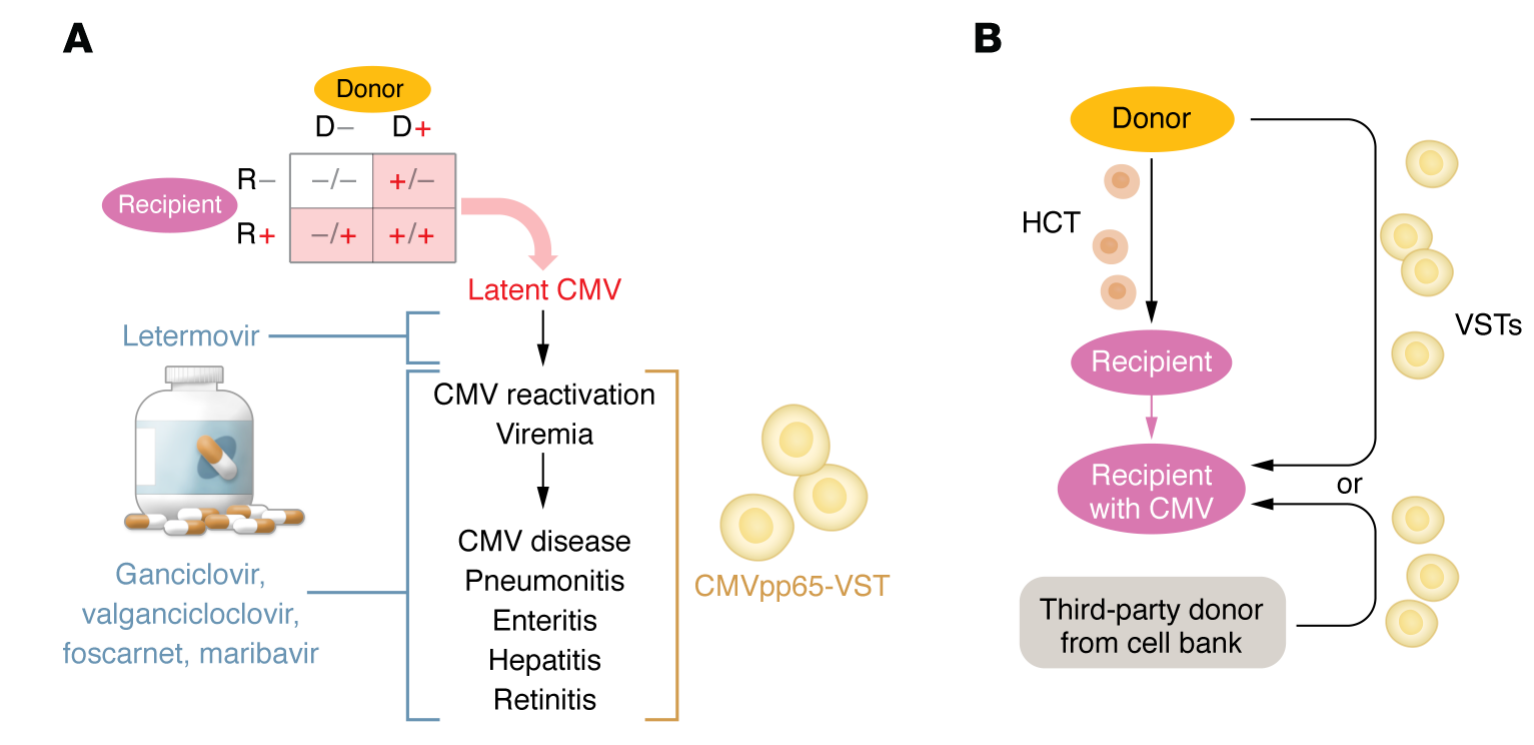
Treatments for CMV therapy after HCT depend on patient status and disease severity. (**A**) CMV-seronegative recipients (R–) of CMV-seronegative donor cells (D–) are at the lowest risk for subsequent CMV reactivation. All other combinations are at increased risk. CMV can reactivate in at-risk patients, resulting in CMV viremia. Untreated, CMV viremia can progress to CMV disease. CMV reactivation can be prevented with letermovir. CMV-specific antivirals can treat CMV reactivation to prevent CMV disease as well as treat CMV disease. CMVpp65-VSTs provide another possible modality for treating CMV reactivation and disease. (**B**) In HCT, recipients initially receive stem cell grafts from allogeneic donors. VSTs can come from the original donor or from a third-party donor distinct from the original donor or recipient.

CMV is a common congenital infection that can cause serious illness in infants, which manifests as deafness and neurodevelopmental delay. In contrast, CMV in immunocompetent adults is often asymptomatic or may present with fever and mononucleosis-like symptoms. CMV can reactivate from a latent state and replicate in immunocompromised patients, such as those who have received allogeneic transplantation or CAR T cell therapy (Figure 1) (5, 6). This reactivation manifests as CMV viremia, and the patient may be febrile, cytopenic, or experience malaise. Unchecked, CMV viremia after allogeneic hematopoietic cell transplantation (HCT) can progress to involve and cause dysfunction in the liver, intestines, retina, and lung. CMV disease of the organs is associated with high mortality; prior to the development of CMV-specific antiviral therapy, 70% of patients with CMV pneumonitis died (7).

Prevention is the therapeutic backbone for CMV infection. Susceptibility to CMV infection is dependent on prior CMV infection, which can be determined by detecting antibodies against CMV. The risk of CMV infection can be reduced in CMV-seronegative patients by choosing CMV-seronegative (rather than seropositive) donors (Figure 1). Because CMV resides in leukocytes, leukocyte filtration of blood products can reduce new infections. Reactivation of latent CMV in high-risk patients can be prevented with letermovir (Figure 1) (8). Early treatment of CMV viremia can stop the progression of CMV reactivation to CMV disease. FDA-approved CMV-specific antiviral agents include ganciclovir/valganciclovir, foscarnet, cidofovir, and recently, maribavir (Figure 1) (9). Despite these preventative and therapeutic methods for reducing the impact of CMV after HCT, persistent CMV reactivation and sometimes disease occur in 28% to 39% of recipients after HCT, thus motivating the development of therapeutics (10, 11).

## CMVpp65-VSTs can eliminate CMV

In this issue of the *JCI*, Prockop et al. (12) present the combined clinical results of three separate phase I or II clinical trials that treated a total of 67 subjects with refractory CMV viremia or organ disease with the adoptive transfer of banked, third-party CMV pp65–sensitized virus-specific T cells (CMVpp65-VSTs). In contrast with donor-derived patient-specific VSTs, each third-party CMVpp65-VST line selected for infusion was (a) restricted by an HLA allele that was shared by the patient and the patient’s HCT donor and (b) shared at least two HLA alleles with the patient (Figure 1). Although Prockop et al. (12) allowed for CMVpp65-VSTs to be administered after failure of one line of therapy, 19 out of 59 subjects had failed three lines of therapy (ganciclovir and/or valganciclovir, foscarnet, and cidofovir and/or brincidofovir), and 20 out of 59 patients had organ involvement and/or organ dysfunction due to CMV infection, indicating a heavily treated and high-risk population. Clinical responses were reported in 38 of 59 (64%) evaluable subjects. Complete response was defined as complete clearance of viremia and biopsy-proven resolution of invasive disease for patients with CMV disease. Partial response was defined as a 2-log, or 100-fold, decrease in CMV viral load and resolution of clinical symptoms related to disease. Overall survival at six months was increased for responders to CMVpp65-VSTs compared with nonresponders (79% versus 29%, *P* < 0.001) (12).

Prockop and authors performed careful correlative studies to elucidate the biological differences between responders and nonresponders. Surprisingly, none of the seven patients receiving VSTs specific for epitopes presented by HLA B35 achieved a response (*P* = 0.001). However, the mechanisms contributing to this lack of response to HLA B35–restricted CMVpp65-VSTs are not well understood. Another factor associated with recipient response to CMVpp65-VSTs was the use of ganciclovir during or before CMVpp65-VST treatment; responses were seen in only 20 of 37 subjects (54%) treated with ganciclovir versus 18 of 22 subjects (82%) treated with other antivirals (but not letermovir). Finally, one concern of third-party VSTs relates to whether the infused cells will adequately expand and persist. The authors confirmed that CMVpp65-VSTs could be detected by functional assays and/or immunophenotyping after infusion of third-party CMVpp65-VSTs in CMV-seropositive recipients transplanted with CMV-seronegative donors. Although the origin of the CMVpp65-VSTs detected after infusion is likely to derive from the third-party donor or possibly from the recipient due to the use of CMV-seronegative HCT donors, chimerism studies were not performed in all cases to definitively identify the source of the CMVpp65-VSTs. Interestingly, serial chimerism analysis in a subset of patients showed that circulating CMVpp65-388–specific T cells after infusion could originate predominantly from the recipient, the stem cell donor, or from the third-party donor. Based on these results, the authors posited that long-term control of CMV in responders may not necessarily be directly from third-party CMVpp65-VSTs; the infused product may somehow stimulate T cells from the stem cell donor or residual recipient T cells to mount a durable anti-CMV response (12).

## Future directions

As with many excellent studies, the results lead to more questions for further exploration. Maribavir, a benzimidazole riboside with anti-CMV activity, was recently approved for patients with refractory CMV after HCT — the very population targeted by these studies (13). Considering maribavir availability, the decreased response with ganciclovir use, and that both ganciclovir and letermovir can inhibit anti-CMV immune responses, presumably by suppressing viral replication and limiting the antigenic exposure of the VSTs, one important question arises: What should be the order of therapy for CMVpp65-VSTs in the current line-up of anti-CMV treatments? (14–16)

The Prockop et al. (12) results also point to potential opportunities for optimizing VSTs. None of the seven subjects treated with CMVpp65-VSTs specific for epitopes presented by HLA B35 responded to therapy, suggesting that these high-risk patients may need alternative strategies. Although endogenous CMV-specific T cells target the dominant protein pp65, they also target intermediate-early 1 protein, albeit at lower frequency. (17) Peptides derived from 151 human CMV–derived open reading frames were immunogenic for T cells from healthy donors (2). Thus, potential epitopes for generating more effective CMV VSTs could be derived from this pool.

## Clinical importance

The Prockop et al. (12) study confirms the safety and therapeutic efficacy of third-party CMVpp65-VSTs, previously reported by other groups (18), and provides further insights into optimizing this therapeutic modality. By using VSTs generated from third-party donors, the applicability of the cells can be extended in principle beyond allogeneic transplantation to other clinical scenarios in which the patient is immunocompromised, such as during chemotherapy, after CAR T cell therapy, and after solid organ transplantation. Prockop et al. (12) and others have shown that CMVpp65-VSTs are an important addition to the armamentarium against CMV. However, further refinements to the modality and determining the circumstances for its application are necessary to optimize the clinical use of VSTs.

## Version 1 05/15/2023

Electronic publication
